# Supplementary material for: Maternal paraben exposure triggers childhood overweight development
Source: Nat Commun. 2020 Feb 11;11:561. doi: 10.1038/s41467-019-14202-1 (PMC7012887; doi:10.1038/s41467-019-14202-1)
Supplement: Supplementary file 1 — Supplementary Information [file 41467_2019_14202_MOESM1_ESM.pdf]

SUPPLEMENTARY INFORMATION FOR

Maternal paraben exposure triggers childhood overweight development

Beate Leppert, Sandra Strunza, Bettina Seiwert, Linda Schlittenbauer, Rita Schlichting, Christiane Pfeiffer, Stefan Röder, Mario Bauer, Michael Borte, Gabriele I. Stangl, Torsten Schöneberg, Angela Schulz, Isabell Karkossa, Ulrike E. Rolle-Kampczyk, Loreen Thürmann, Martin von Bergen, Beate Escher, Kristin Junge, Thorsten Reemtsma, Irina Lehmann, Tobias Polte

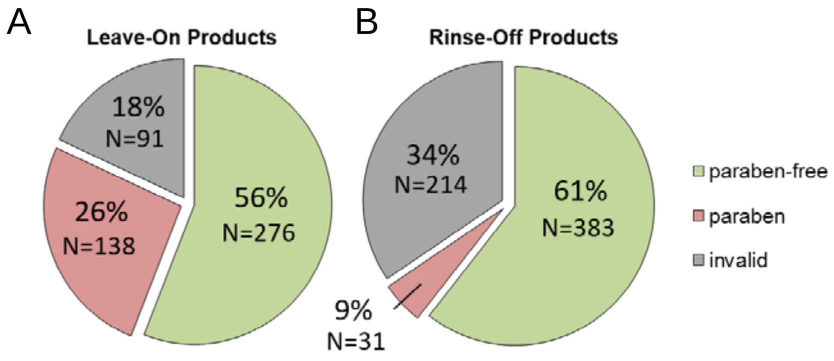

Supplementary Figure 1: Distribution of paraben containing cosmetic product application in the LINA study. Percentage of participants (n = 629) that named at least one leave-on (A) or rinse-off (B) cosmetic product that contains parabens or used paraben-free products only. Insufficiently indicated cosmetics that could not be assigned to a specific brand/product were excluded from further analyses (invalid).

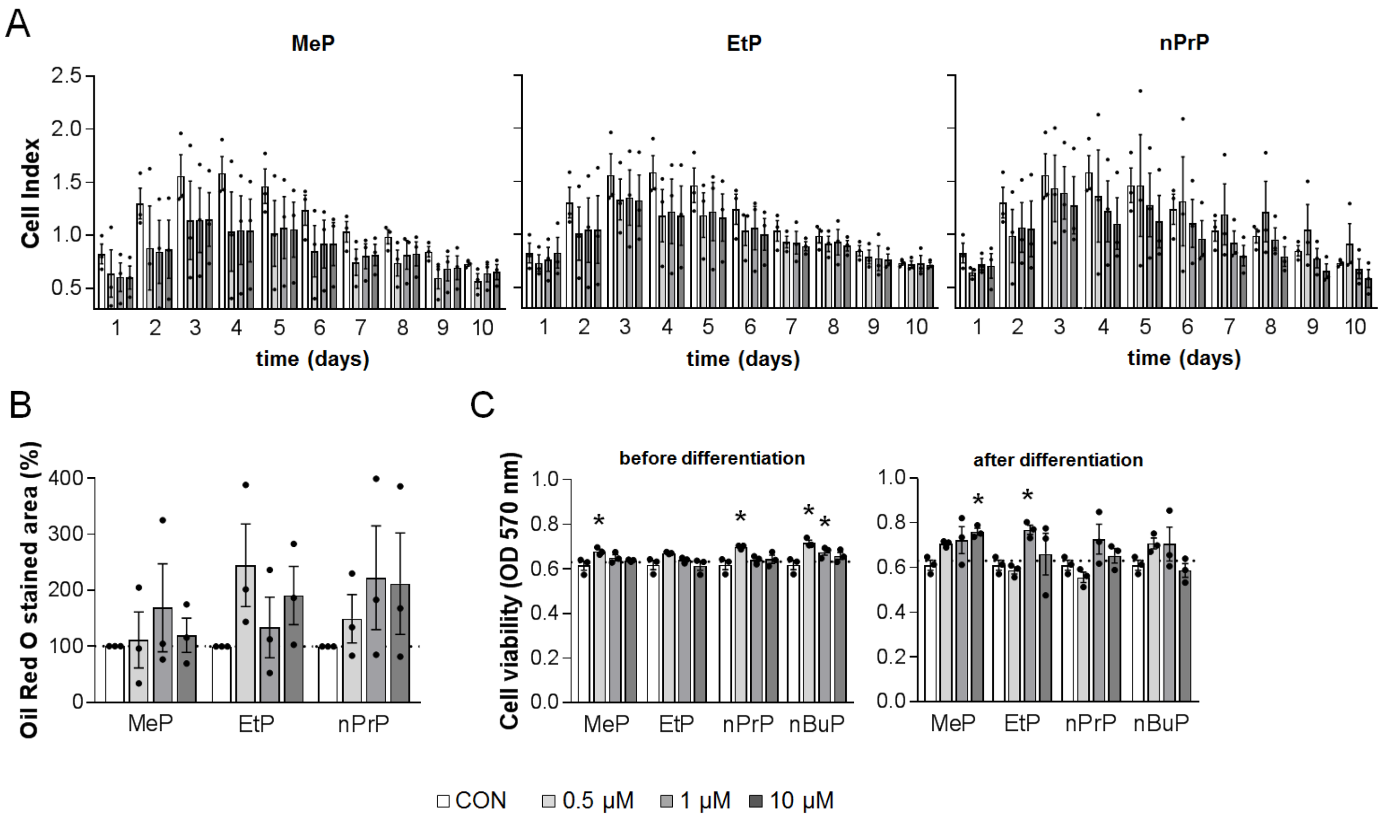

Supplementary Figure 2: Adipogenesis under paraben exposure. (A) Real-time monitoring of adipocyte differentiation from human MSCs with exposure to 0.5 - 10 µM parabens. Human mesenchymal stem cells were differentiated to adipocytes for 10 days under exposure to 0.5 - 10 µM of either methylparaben (MeP), ethylparaben (EtP), and n-propylparaben (nPrP). Shown are normalised cell index values (xCELLigence System) over 10 days of differentiation (mean ± SEM, n = 3). (B) Triglyceride storage of adipocytes assessed via Oil Red O staining. (C) Impact of paraben exposure on cell viability with regard to the control. Shown are means ± SEM for n = 3 experiments. with *P < 0.05, ANOVA. Source data are provided as a Source Data file.

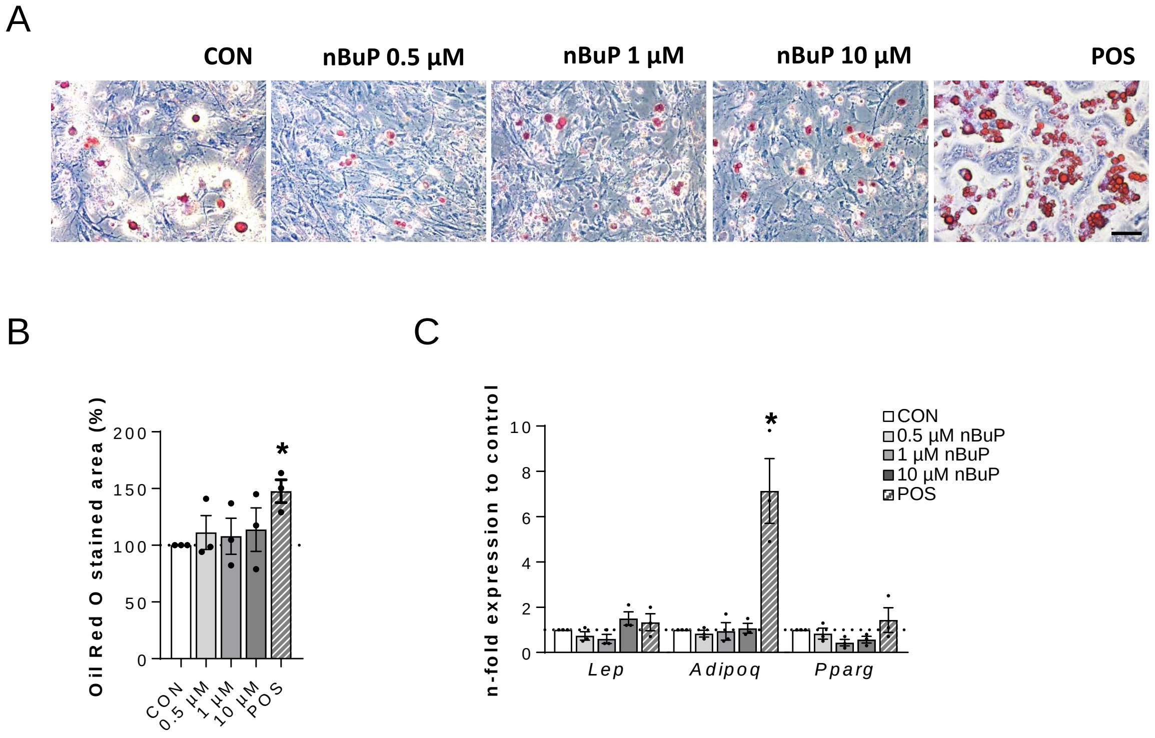

Supplementary Figure 3: Effect of nBuP exposure on murine adipocyte differentiation. In vitro adipocyte differentiation from murine mesenchymal stem cells (MSC) in the presence of nBuP and rosiglitazone (POS). (A) Representative Oil Red O stained pictures after differentiation, scale bar: 100 µm (B) Triglyceride storage of adipocytes assessed via Oil Red O staining. (C) Gene expression of leptin (Lep), adiponectin (Adipoq) and the transcription factor peroxisome proliferator-activated receptor gamma (Pparg). Data are expressed as mean ± SEM of n = 3 experiments. *$P < 0.05$, unpaired t-test. Source data are provided as a Source Data file.

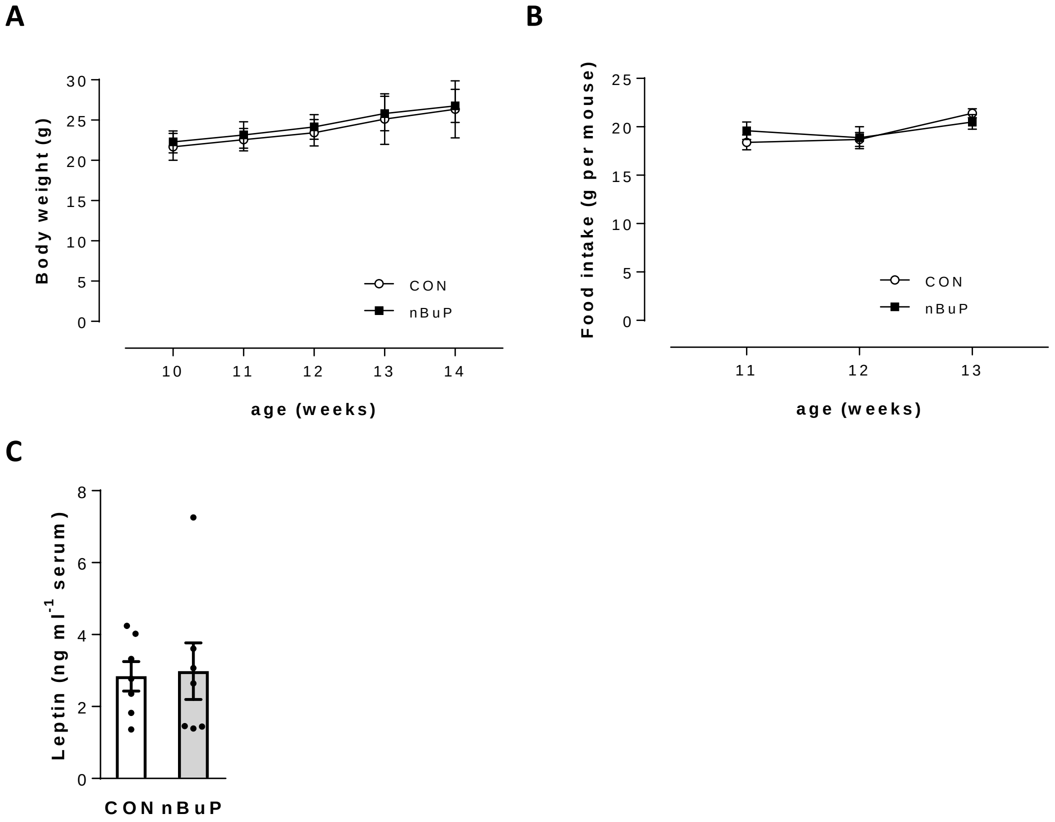

Supplementary Figure 4: Effect of nBuP exposure to adult mice on weight development, food intake and leptin serum levels.

(A) Bodyweight development (n = 13), (B) food intake (n = 7) and (C) leptin serum levels (n = 7) are shown from female mice exposed to nBuP. Data are expressed as mean ± SEM. *P < 0.05, unpaired t-test. Source data are provided as a Source Data file.

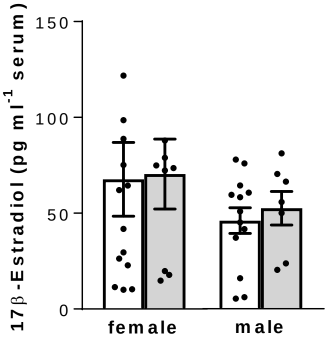

Supplementary Figure 5: Effect of perinatal nBuP exposure on serum 17ß-Estradiol level in the offspring.
Level of 17ß-Estradiol measured in serum of female and male offspring. Data are expressed as mean ± SEM, CON: n = 13, nBuP: n = 7. *P < 0.05, unpaired t-test. Source data are provided as a Source Data file.

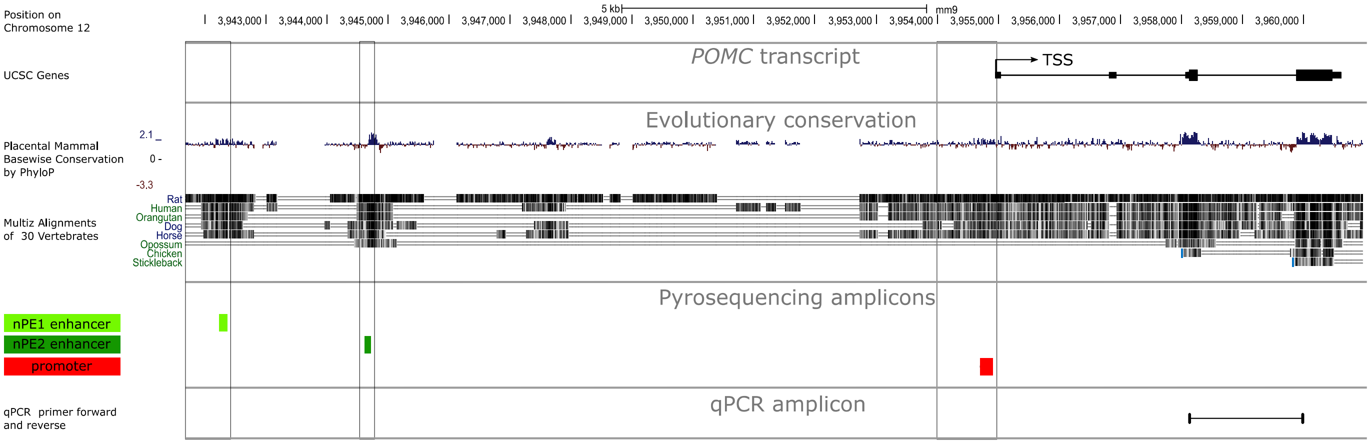

Supplementary Figure 6: Genomic location of *Pomc* promoter and enhancers nPE1, and nPE2. Genomic positions of the PCR amplicons used for DNA-methylation assessment by pyrosequencing of *Pomc* regulatory regions are depicted as red and green bars. The promoter region (highlighted in light red, chr12:3,954,951-3,953,951) is defined as 1 kbp upstream of the *Pomc* transcription start site (TSS). The genomic regions of enhancer nPE1 (chr12:3941500-3942350, highlighted in light green) and nPE2 (chr12:3944545-3944766, highlighted in dark green) were determined based on Drouin *et al*., 2016, Rubinstein *et al*., 2017 and Langlais *et al*., 2011. UCSC gene and phylogenetic conservation tracks obtained from the UCSC genome browser (NCBI37/mm9 mouse reference genome) demonstrating highly conserved enhancer and promoter regions of the *Pomc* gene across mammals. In the bottom, genomic position of qPCR primer is depicted.

A

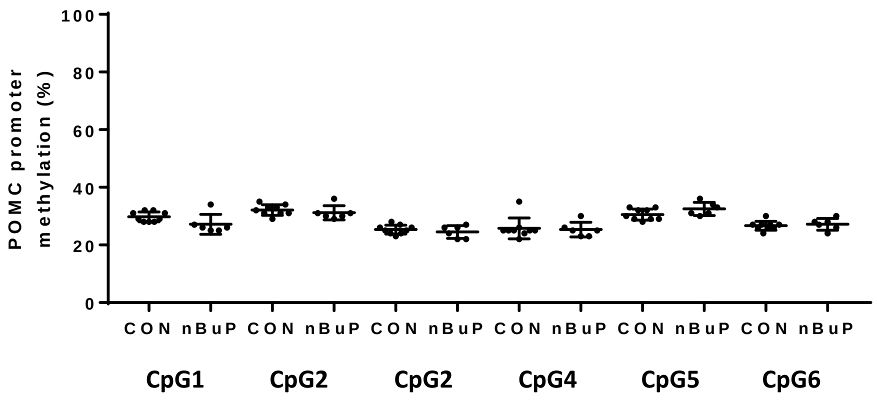

B

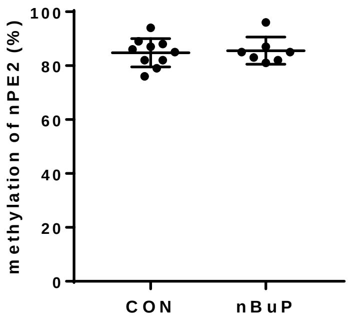

Supplementary Figure 7: Perinatal nBuP exposure did not alter DNA methylation of the pro-opiomelanocortin (POMC) promotor and the neuronal POMC enhancer nPE2 in the hypothalamus of female offspring.
(A) DNA methylation of the neuronal POMC promotor (chr12:3954951-3953951) were analysed from the 12-weeks-old female offspring (CON: n = 9, nBuP: n = 6). Values for DNA methylation in both groups are shown for CpG1 (chr12:3954890), CpG2 (chr12:3954873), CpG3 (chr12:3954862), CpG4 (chr12:3954852), CpG5 (chr12:3954849) and CpG6 (chr12:3954842). (B) The DNA methylation level of the neuronal POMC enhancer of nPE2 (chr12:3944545-3944766) was investigated in the hypothalamus of the 12-weeks-old female offspring (CON: n = 10, nBuP: n = 7) and pictured for CpG2 (cg3944695). Data are expressed as mean ± SEM. *P < 0.05, unpaired t-test. Source data are provided as a Source Data file.

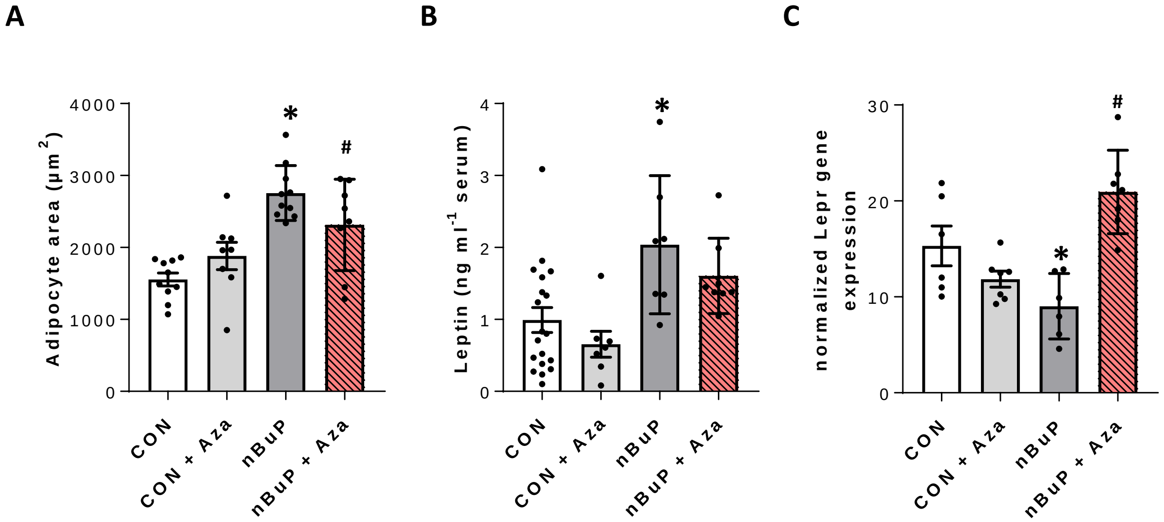

Supplementary Figure 8: Effect of the DNA methyl-transferase inhibitor Aza.

After treatment of F1 mice with the DNA methyl-transferase inhibitor Aza adipocyte area (A), leptin serum levels (B) and leptin receptor (Lepr) expression in the hypothalamus (C) were evaluated. Data are expressed as mean ± SEM, n = 7 animals per group; *p < 0.05 for nBuP *vs.* CON, #P < 0.05 for nBuP + Aza *vs*. nBuP, unpaired t-test. Source data are provided as a Source Data file.

Supplementary Table 1: Study characteristics of the total study cohort and the analysed sub-cohort with maternal urinary paraben measurements available.

	**Entire LINA cohort** n (%), n = 629[a]	**Analysed sub-cohort** n (%), n = 223	χ^2-*test*[b]
**Gender of the child**			
Female	302 (48.0)	115 (51.6)	0.361
Male	327 (52.0)	108 (48.4)	
**Birth weight**			
≤3000g	123 (19.6)	42 (18.8)	0.958
>3000-3500g	242 (38.5)	84 (37.7)	
>3500-4000g	192 (30.6)	69 (30.9)	
>4000g	71 (11.3)	28 (12.6)	
**Gestational week at delivery**			
< 37 week s	25 (4.0)	9 (4.0)	0.830
37 – 40 weeks	389 (62.0)	143 (64.1)	
>40 weeks	214 (34.0)	71 (31.8)	
**Maternal age at pregnancy**			
< 31 years	305 (48.5)	118 (52.9)	0.256
≥ 31 years	324 (51.5)	105 (47.1)	
**Smoking during pregnancy**			
Never	534 (84.9)	198 (88.8)	0.245
Occasionally	47 (7.4)	15 (6.7)	
Daily	48 (7.6)	10 (4.5)	
**Parental school education**[c]			
Low	16 (2.5)	3 (1.4)	0.281
Intermediate	144 (22.9)	43 (19.3)	
High	469 (74.6)	177 (79.4)	
**Siblings**			
Yes	208 (33.4)	81 (36.3)	0.436
No	414 (66.6)	142 (63.7)	
**Breastfeeding exclusive**			
Never	26 (4.1)	9 (4.0)	0.340
1-3 month	112 (17.8)	31 (13.9)	
1-6 month	190 (30.2)	79 (35.4)	
1-12 month	254 (40.4)	104 (46.6)	

[a] n may be different from 629 due to missing data

[b] calculated using the chi squared test for cross relationship

[c] low = 8 yrs of schooling (‘Hauptschulabschluss`); intermediate = 10 yrs of schooling (`Mittlere Reife`); high = 12 yrs of schooling or more (`(Fach-)hochschulreife’)

Supplementary Table 2: Maternal urinary paraben concentrations (µg l^{-1}) of the LINA study cohort for all participants with valid paraben measurements and (A) weight associations at birth (B) weight associations in early to mid-childhood

(A) n=496	**MeP**	**EtP**	**nPrP**	**iBuP**	**nBuP**
Mean	139	14.5	30.2	1.8	6.6
Median	39.2	2.3	5.0	0.2	0.7
Min	0.25	<0.05	<0.05	<0.05	<0.05
Max	3680	269	1182	97.2	269

(B) n=223	**MeP**	**EtP**	**nPrP**	**iBuP**	**nBuP**
Mean	136.2	14.3	29.3	1.8	6.9
Median	39.9	2.6	4.4	0.2	0.8
Min	0.25	<0.05	<0.05	<0.05	<0.05
Max	3680	195	898	46.4	269

Supplementary Table 3: Reporter gene assays. Butylparaben was tested in five reporter gene cell lines. Concentration-response curves were plotted for cytotoxicity ($IC_{10,}$ μM)) and activation ($EC_{10,}$ μM) of the reporters.

Reporter gene assay	IC_{10} cytotoxicity	EC_{10} activation
Peroxisomal proliferator-activated receptor (PPARgamma)	4.7	no effect up to IC_{10}
Androgen receptor (AR)	4.0	no effect up to IC_{10}
Estrogen receptor-α (ER-α)	4	0.48±0.02
Progesterone receptor (PR)	6.5	no effect up to IC_{10}
Glucocorticoid receptor (GR)	8	no effect up to IC_{10}

Supplementary Table 4: Primers used for qPCR studies.

Gene	Forward (5´-3´)	Reverse (5´-3´)
Human		
PGK1	GACCGAATCACCGACCTCTC	AGCAGCCTTAATCCTCTGGT
GUSB	GTCTGCGGCATTTTGTCGG	CACACGATGGCATAGGAATGG
PPARG	TTACGCCTCGGTGTTTAGGG	TGGTCATTTCGTTAAAGGCTGA
LEP	TTTCACACACGCAGTCAGTC	GTGGAGCCCAGGAATGAAGT
ADIPOQ	TGACCAGGAAACCACGACTC	AGGACCAATAAGACCTGGATCTC
Mouse		
Pparg	CCACCAACTTCGGAATCAGCT	TTTGTGGATCCGGCAGTTAAGA
Glut4	TGTCGCTGGTTTCTCCAACTG	CCATACGATCCGCACATACTG
Insr	GCGGCCTCATCTTCTTCACT	AACTGAAGTCCAGGTTCATATAGTCAGA
Agrp	CTCCACTGAAGGGCATCAGAAG	ACTCAGCACCTCCGCCAAAG
Lepr	TGAATTTCCAAAAGCCTGAAACA	CCAGAAGAAGAGGACCAAATATCAC
Mc4r	GTCAGGCGTCCTCTTCATCA	AGGTAATCGCCCCCTTCA
Pomc	GGAAGATGCCGAGATTCTGC	TCCGTTGCCAGGAAACAC
U6	AACGCTTCACGAATTTGCGT	CTCGCTTCGGCAGCACA
Gusb	GTGGTATGAACGGGAAGCAAT	AACTGCATAATAATGGGCACTGT
Actb	GTGACGTTGACATCCGTAAAGA	GCCGGACTCATCGTACTCC

Supplementary Table 5: Effect of nBuP and AZA treatment on offspring bodyweight and food intake according to Figure 4D. Effect estimates were derived by GEE models with exchangeable correlation matrix. Changes over time were estimated by including interaction term of each exposure condition with time (week). Models are shown for control (A) and BuP_AZA treated (B) as reference group.

**A. To control**	**Weight in g**			**Food intake in g**		
	β (g)	SE	p	β (g)	SE	p
Intercept	5.23	0.34	<2x10-16	20.34	0.76	<2x10-16
Week	1.57	0.04	<2x10-16	-0.28	0.10	0.006
BuP	2.97	0.51	5x10-9	-1.54	1.31	0.242
Con_AZA	2.13	0.53	6x10-5	1.23	1.15	0.284
BuP_AZA	2.48	0.58	3x10-5	2.15	1.14	0.061
Interactions with time (week of assessment)						
BuP	-0.03	0.07	0.670	0.47	0.16	0.004
Con_AZA	-0.14	0.06	0.044	-0.06	0.15	0.678
BuP_AZA	-0.20	0.08	0.009	-0.14	0.15	0.325

**B. To BuP_AZA**	**Weight in g**			**Food intake in g**		
	β (g)	SE	p	β (g)	SE	p
Intercept	7.71	0.48	<2x10-16	22.49	0.86	<2x10-16
Week	1.37	0.06	<2x10-16	-0.43	0.10	3.6x10-5
BuP	*0.48*	*0.62*	*0.433*	*-3.68*	*1.37*	*0.007*
Con_AZA	-0.35	0.64	0.587	-0.91	1.22	0.454
Con	-2.48	0.59	2.5x10-5	-2.15	1.15	0.061
Interactions with time (week of assessment)						
BuP	*0.17*	*0.08*	*0.028*	*0.62*	*0.16*	*0.0002*
Con_AZA	0.06	0.08	0.428	0.09	0.15	0.571
Con	0.20	0.08	0.009	0.15	0.15	0.325
