# Supplementary material for: Maternal paraben exposure triggers childhood overweight development
Source: Nat Commun. 2020 Feb 11;11:561. doi: 10.1038/s41467-019-14202-1 (PMC7012887; doi:10.1038/s41467-019-14202-1)
Supplement: Supplementary file 3 — Reporting Summary [file 41467_2019_14202_MOESM3_ESM.pdf]

nature research

Corresponding author(s): NCOMMS-18-19701C

Reporting Summary

Nature Research wishes to improve the reproducibility of the work that we publish. This form provides structure for consistency and transparency in reporting. For further information on Nature Research policies, see Authors & Referees and the Editorial Policy Checklist.

Statistical parameters

When statistical analyses are reported, confirm that the following items are present in the relevant location (e.g. figure legend, table legend, main text, or Methods section).

n/a	Confirmed	
☐	☒	The exact sample size (*n*) for each experimental group/condition, given as a discrete number and unit of measurement
☐	☒	An indication of whether measurements were taken from distinct samples or whether the same sample was measured repeatedly
☐	☒	The statistical test(s) used AND whether they are one- or two-sided<br>*Only common tests should be described solely by name; describe more complex techniques in the Methods section.*
☐	☒	A description of all covariates tested
☐	☒	A description of any assumptions or corrections, such as tests of normality and adjustment for multiple comparisons
☐	☒	A full description of the statistics including central tendency (e.g. means) or other basic estimates (e.g. regression coefficient) AND variation (e.g. standard deviation) or associated estimates of uncertainty (e.g. confidence intervals)
☐	☒	For null hypothesis testing, the test statistic (e.g. *F*, *t*, *r*) with confidence intervals, effect sizes, degrees of freedom and *P* value noted<br>*Give P values as exact values whenever suitable.*
☒	☐	For Bayesian analysis, information on the choice of priors and Markov chain Monte Carlo settings
☒	☐	For hierarchical and complex designs, identification of the appropriate level for tests and full reporting of outcomes
☐	☒	Estimates of effect sizes (e.g. Cohen's *d*, Pearson's *r*), indicating how they were calculated
☐	☒	Clearly defined error bars<br>*State explicitly what error bars represent (e.g. SD, SE, CI)*

Our web collection on statistics for biologists may be useful.

Software and code

Policy information about availability of computer code

Data collection	The content of parabens in the named cosmetic products was assessed between April and July 2016 via the TOXFOX app for iOS from the “Friends of the earth Germany” (“Bund für Umwelt und Naturschutz Deutschland”).
Data analysis	LINA study data were evaluated by STATISTICA for Windows, Version 12 (Statsoft Inc.,USA) and STATA version 15.1 (StataCorp LLC, USA). Mouse data were analysed with GraphPad Prism 7 or MS Excel. GEE models were evaluated with R version 3.5.1.

For manuscripts utilizing custom algorithms or software that are central to the research but not yet described in published literature, software must be made available to editors/reviewers upon request. We strongly encourage code deposition in a community repository (e.g. GitHub). See the Nature Research guidelines for submitting code & software for further information.

Data

Policy information about availability of data

All manuscripts must include a data availability statement. This statement should provide the following information, where applicable:

- Accession codes, unique identifiers, or web links for publicly available datasets
- A list of figures that have associated raw data
- A description of any restrictions on data availability

All data generated or analysed during this study are included in this published article (and its supplementary information files) or could be requested from the

corresponding author.

Field-specific reporting

Please select the best fit for your research. If you are not sure, read the appropriate sections before making your selection.

☒ Life sciences ☐ Behavioural & social sciences ☐ Ecological, evolutionary & environmental sciences

For a reference copy of the document with all sections, see nature.com/authors/policies/ReportingSummary-flat.pdf

Life sciences study design

All studies must disclose on these points even when the disclosure is negative.

Sample size	Either for the human cohort, nor for the cell culture experiments a sample size calculation was performed.<br>For the human cohort data, all samples available within the entire LINA study (n=629 children and 622 mothers) that had complete information with respect to the research question were used for analyses and meassurements.<br>For cell culture (n= 3 experiments, each including all experimental conditions in duplicates) standardized sample sizes were used.<br>For mouse experiments an analysis with G-Power (3.1) was performed based on values received from earlier studies with a similar approach (Bisphenol A and weight).
Data exclusions	Indicated cosmetic products were searched for their paraben content with the TOXFOX app (described in Methods) and categorised in leave-on and rinse-off products. Since exposure time is obviously very short, and TOXFOX indicated only 31 out of 414 rinse-off products as paraben containing (Supplementary Figure S1), only leave-on products with a high exposure time and high body area coverage were considered for further analyses regarding overweight development. In mouse experiments data from mice showing unusual behavior or signs of pain/illness were excluded.
Replication	For the human cohort data, no replacation was performed. Data from cell culture experiments are presented from n= 3 experiments, each including all experimental conditions in duplictes. Mouse experiments included groups of 4-6 mice/cage and were performed at least two times.
Randomization	For the human cohort data, participants were categorized not randomly into outcome positive "overweight" or outcome negative "non overweight" according to their BMI assessment and WHO reference data. Further, and also not randomly, participants were grouped objectively according to their exposure or non-expusure to parabens. Data from cell culture experiments were presented from n= 3 experiments, each including all experimental conditions in duplicates. Within the mouse experiments no randomization was carried out.
Blinding	For human cohort data, investigators were blinded to the identity of the study participants. Due to the experimental setting of the validation experiments, investigators of cell culture and mouse experiments selectively tested exposed vs non-exposed conditions in a small and non blinded set up.

Reporting for specific materials, systems and methods

Materials & experimental systems

n/a	Involved in the study	
☐	☒	Unique biological materials
☒	☐	Antibodies
☐	☒	Eukaryotic cell lines
☒	☐	Palaeontology
☐	☒	Animals and other organisms
☐	☒	Human research participants

Methods

n/a	Involved in the study	
☒	☐	ChIP-seq
☒	☐	Flow cytometry
☒	☐	MRI-based neuroimaging

Unique biological materials

Policy information about availability of materials

Obtaining unique materials	Unique samples from the human cohort study LINA were used for urine analyses. Available amounts of samples from cohort study participants are generally limited (ml blood or ml urine that were donated by each participant) and unique regarding time points of sample assessment.

Eukaryotic cell lines

Policy information about cell lines

Cell line source(s)	Adipocyte derived mesenchymal stem cells (from ATCC).
Authentication	Authenticated adipocyte derived mesenchymal stem cells were commercially available from ATCC.
Mycoplasma contamination	Mesenchymal stem cells were negative for mycoplasma contamination according to the ATCC datasheet.
Commonly misidentified lines (See ICLAC register)	No commonly misidentified lines were used.

Animals and other organisms

Policy information about studies involving animals; ARRIVE guidelines recommended for reporting animal research

Laboratory animals	Female BALB/cByJ mice (6-8 weeks of age) were obtained from the Elevage Janvier Laboratory (Le420 Genest St Isle, France). Animal protocols used in this study were approved by the Committee on Animal Welfare of Saxony/Leipzig (Permit Number: TVV01/15).
Wild animals	The study did not involve wild animals/animals captured in the field.
Field-collected samples	The study did not involve samples collected from the field/from wild animals.

Human research participants

Policy information about studies involving human research participants

Population characteristics	Standardised self-administered questionnaires were collected annually starting in pregnancy, assessing general information about personal lifestyle, housing and environmental conditions and disease state. General characteristics of the study participants are shown in supplementary Table S1.
Recruitment	The German prospective birth cohort LINA (Lifestyle and environmental factors and their Influence on Newborns Allergy risk) recruited 622 mothers (629 children) at 34 weeks of gestation between May 2006 and December 2008 in Leipzig, Germany. Mothers with severe immune or infectious diseases during pregnancy were excluded from the study. The study was approved by the Ethics Committee of the University of Leipzig (file ref # 046-2006, 160-2008, 160b/2008, 144-10-31052010, 113-11-18042011, 206-12-02072012, #169/13-ff, #150/14-ff). There was no self selection bias.
